# Impact of COVID-19 on physical activity patterns in non-professional populations in Asia: a mini review of pre-, during, and post-pandemic periods

**DOI:** 10.3389/fpubh.2025.1604185

**Published:** 2025-07-15

**Authors:** Zonglin Jiang, Qianhui Zhou, Haolu Shu, Linjing Jiang

**Affiliations:** ^1^Department of Physical Education, Changchun Dongfang Vocational College, Jilin, China; ^2^Graduate School of Sport Sciences Waseda University, Tokorozawa, Saitama, Japan; ^3^Faculty of Sport Sciences, Waseda University, Tokyo, Japan; ^4^Institutes of Innovation for Future Society, Nagoya University, Nagoya, Japan

**Keywords:** COVID-19 pandemic, exercise habits, physical activity, exercise patterns, public health

## Abstract

This mini-narrative review examines the impact of the COVID-19 pandemic on physical activity (PA) patterns across Asian countries, including regions such as Hong Kong, South Korea, Japan, and Southeast Asia. Pre-pandemic (before 2019), Asia experienced gradually increasing PA participation rates, characterized predominantly by outdoor activities, gym workouts, and organized group exercises, driven by growing fitness awareness and the availability of facilities for exercise. During the pandemic (2020–2022), widespread declines in regular exercise occurred due to restrictions, causing a substantial shift toward indoor, home-based, and online-based PA. Low PA adversely affects cardiovascular health, immune function, obesity, metabolic conditions, and psychological well-being. Although home-based exercise modalities partially mitigated these impacts, their effectiveness remained limited compared to pre-pandemic routines. In the post-pandemic period (2022–2025), PA in Asia partially recovered, with some regions, such as Hong Kong and South Korea, reporting PA levels surpassing pre-pandemic baselines due to widespread adoption of hybrid exercise models. This recovery has fostered lasting changes toward hybrid exercise models, combining traditional and digital modalities, resulting in positive health outcomes across the cardiovascular, immune, metabolic, and psychological domains. Future public health strategies should emphasize flexible, diverse, and accessible exercise options, and further research should explore the sustainability and implications of these evolving exercise behaviors.

## 1 Introduction

The COVID-19 pandemic and associated restrictions triggered a global decline in physical activity (PA) participation (1, 2). This decline is evidenced by fewer daily steps, reduced moderate-to-vigorous PA, and increased sedentary time (3). Community PA initiatives were also severely disrupted in Asia, as many Asian countries enforced lockdowns, social distancing, facility closures, and self-isolation measures (4–7). These measures particularly affected the general population in Asia, where non-professional individuals experienced some of the most significant lifestyle disruptions. While most existing studies have concentrated on elite athletes, research addressing the general, non-professional population, who experienced broader and more widespread impacts, remains limited. Several studies have also examined PA during the pandemic in non-professional populations (8, 9), and some participants have self-reported that their activity levels were affected by pandemic-related restrictions. Moreover, most available research is cross-sectional and global in scope (10–14), providing limited insight into how PA has changed in Asia over time. However, longitudinal data tracking PA across the pre-pandemic, pandemic, and post-pandemic phases are lacking. In particular, the psychological consequences of both PA reduction and recovery, such as chasnges in motivation, well-being, and habit formation remain understudied (15, 16). Therefore, reviewing pandemic-era studies, especially from a temporal perspective, may help provide a more comprehensive understanding of how PA behaviors among non-professional populations evolved throughout different phases of the pandemic and identify pandemic-specific factors that may have influenced these patterns.

Some recent Asia-specific studies have emerged, indicating that virtual reality interventions have improved adolescent fitness in Indonesia (11), hybrid PA learning models have been implemented for students (17), and regional disparities in inactivity during the pandemic have been documented across age groups and countries (18–22). These findings highlight the need for a mini-review focusing on the temporal evolution of PA behaviors in Asia throughout the pandemic timeline.

This mini-review examines shifts in exercise patterns among Asia's general population across three periods: pre-pandemic (before 2019), pandemic (2020–2022), and post-pandemic (after 2023). Specifically, it analyzes changes in exercise modes–outdoor, gym-based, group-based, indoor home-based, and hybrid activities–and evaluates the impacts of these shifts on physical health (e.g., cardiovascular health, immune function, obesity, and metabolic conditions) and psychological well-being (e.g., motivation, mental health, and habit formation). The insights from this review aim to guide public health strategies and help the fitness industry adapt to evolving exercise preferences.

## 2 Methods

A mini-narrative review approach was used to examine PA patterns in Asia before, during, and after the COVID-19 pandemic. A comprehensive literature search of the PubMed and Web of Science databases was conducted for relevant studies published between 2016 and 2025, restricted to English-language publications only. The search strategy combined keywords such as “physical activity”, “exercise”, “COVID-19”, “pandemic”, “Asia”, and specific country names (e.g., China, Japan, South Korea, and Indonesia) to identify pertinent articles.

The inclusion criteria were as follows: (1) published between 2016 and 2025; (2) focused on non-professional (general population) PA; and (3) reported PA trends or behavioral impacts pertaining to the pre-pandemic, pandemic, or post-pandemic periods in Asian populations. Studies focusing on elite athletes or those not relevant to general population PA were excluded. These studies were descriptively synthesized (qualitative narrative synthesis) to highlight changes in PA behaviors across the specified periods.

## 3 Pre-pandemic (before 2019)

Prior to the COVID-19 outbreak, Asia experienced a gradual increase in PA participation rates, although physical inactivity remained a persistent challenge to public health (23, 24). According to a 2018 study, approximately 27.5% (range: 25.0–32.2%) of the global population is categorized as physically inactive, which is higher in Asian countries, and this proportion has been relatively stable since 2001 (25). Notably, affluent regions in Asia exhibit higher rates of sedentary behavior among adolescents than other regions globally (25–27). Exercise motivation during this period typically centered on physical health improvement, fitness maintenance, and social engagement (28). Consequently, the main forms of exercise are traditional outdoor activities, such as jogging in parks and participating in community sports, as well as indoor fitness, such as gym workouts and yoga classes, which are also increasing (27). Overall, in Asia, there was an upward trend in people's participation in PA prior to the outbreak, and awareness of the health benefits of exercise has been increasing owing to the growing availability of fitness facilities and community-based sports activities. This period provides an important benchmark for assessing the impact of PA trends after the COVID-19 outbreak.

## 4 During the pandemic (2020–2022)

During the COVID-19 pandemic, daily routines were profoundly disrupted, leading to a substantial decline in exercise habits among many individuals. Long-standing physical activities and hobbies were abruptly suspended, making it difficult for even highly motivated individuals to maintain their exercise habits. Populations dependent on organized group activities or gym facilities, such as older adults participating in community programs or frequent gym-goers, faced heightened challenges in preserving their habitual exercise. Some studies suggest that the shift in exercise behaviors and the rise in sedentary lifestyles during lockdowns contributed to an increased risk of chronic and lifestyle-related diseases (29, 30). During this period, search queries for “home exercise” also rushed into the tens of millions, highlighting the widespread enthusiasm for adapting to the new circumstances (31, 32). Consequently, living rooms and rooftops were transformed into makeshift gyms as people engaged in indoor activities such as bodyweight workouts, yoga, dancing, and rope skipping (32). Online platforms have also experienced rapid growth, with virtual fitness classes and social media challenges enabling people to participate ingroup exercisese remotely (32). Even though individuals exercised alone at home, these online services provided essential social support and motivation to continue exercising.

This section examines the impact of altered exercise patterns during the COVID-19 pandemic on both physical health, specifically cardiovascular health, immune function, obesity, and metabolic conditions, and psychological wellbeing (Table 1).

**Table 1:** Impacts of reduced physical activity (PA) during the COVID-19 pandemic (2020–2022).

**Health domain**	**Relationship between PA and health**	**Changes during the pandemic**	**References**
Physical health: Cardiovascular Health	Regular PA improves cardiovascular function, lowers morbidity, mortality, and enhances endocrine function.	Decline in outdoor and group-based PA adversely affected blood glucose, blood pressure, and triglycerides.	(29, 33–35)
Physical health: Immune Function	Moderate PA boosts immune function, reducing infection risk and severity.	Reduced PA potentially weakened immunity, increasing susceptibility and severity of COVID-19 symptoms; older adult particularly vulnerable.	(36–38)
Physical health: Obesity and Metabolic Conditions	Regular PA controls weight, improves insulin sensitivity, and prevents metabolic disorders.	Significant decrease in PA and increased sedentary behavior led to notable weight gain, higher BMI, and poorer diabetic control.	(29, 34)
Psychological wellbeing	PA alleviates depressive and anxiety symptoms, enhances cognitive function, reduces loneliness, and improves mood through social interaction and neurotransmitter regulation.	Reduced PA exacerbated psychological distress, stress, loneliness, depressive symptoms, and negatively impacted neurotransmitter synthesis.	(29, 37, 39–43)

### 4.1 physical health

#### 4.1.1 Cardiovascular health

Regular physical exercise is widely recognized for promoting cardiovascular health, reducing the mortality and morbidity associated with heart diseases, and improving overall quality of life. Early in the pandemic, the World Health Organization (WHO) emphasized the importance of maintaining PA during self-isolation (WHO guidance). Pre-pandemic studies (33) and emerging evidence during the pandemic (34, 35) support the notion that regular exercise can enhance endocrine function and regulate protective enzymes, thereby bolstering resistance to complications after infection.

However, multiple reports have indicated that outdoor and group-based physical activities diminished sharply during the pandemic. While some people compensated with indoor or home-based exercise, it often failed to offset the shortfall in overall PA (29, 34). Consequently, markers such as blood glucose, blood pressure, and triglyceride levels were adversely affected, posing potential risks to cardiovascular health.

#### 4.1.2 Immune function

Several studies have highlighted that reduced PA levels and altered exercise modalities during the pandemic influenced the immune system of the general population, potentially affecting both COVID-19 susceptibility and symptom severity. However, the existing evidence is not entirely consistent. Some researchers have proposed the so-called “Open Window Theory”, suggesting that intense exercise might transiently increase the infection risk and amplify symptoms. Conversely, other investigations underscore the immune-protective benefits of moderate, regular PA, particularly in older adults, who may experience reduced infection rates and milder symptoms owing to enhanced immune regulation (36–38). Despite ongoing debate, older individuals with weakened immune function face notable risks when their activity levels decline during a pandemic. In such cases, safe indoor exercise routines are crucial for mitigating the threat of COVID-19 and maintaining essential immune responses. This highlights the need to adapt PA guidelines to meet the unique requirements of vulnerable populations, emphasizing strategies that preserve immune function and promote overall health in restrictive or high-risk environments.

#### 4.1.3 Obesity and metabolic conditions

Pandemic-related lifestyle changes, such as reduced movement and prolonged sedentary periods, have led many individuals to abandon or drastically reduce their usual exercise regimens. Even those with strong motivation found it challenging to sustain their workouts without access to group activities or gym facilities. In Japan, longitudinal research has revealed a marked drop in exercise adherence by late 2020, especially among older women and high-income populations. Reduced energy expenditure, coupled with stress-induced overeating, contributed to notable weight gain in several communities; the prevalence of overweight/obesity in Japanese men rose from 22.2% to 26.6%, and in women from 9.3% to 10.8%, within a single year.

Similar trends have been reported, with drops in PA during lockdown cited as a primary driver of increased BMI (29, 34). A scoping review of Asian populations found that pandemic-driven declines in PA and increases in sedentary behavior were linked to weight gain, poorer diabetic control, and other negative metabolic outcomes. Nonetheless, a minority who managed to maintain or initiate new at-home exercise routines tended to exhibit more favorable metabolic indicators and health profiles.

### 4.2 Psychological wellbeing

While many mental health challenges arising during the COVID-19 pandemic can be directly attributed to lockdown measures and prolonged home confinement, diminished PA appears to have further exacerbated psychological distress. Previous studies underscore the essential role of regular exercise in preserving mental health and cognitive function, demonstrating that consistent PA can alleviate depressive symptoms (39), reduce anxiety (40), and enhance cognitive performance in older adults (41). In addition, engaging in group-based activities may offer valuable social interaction, thereby mitigating loneliness (37). From a physiological standpoint, sustained reductions in PA can disrupt the synthesis of key neurotransmitters (e.g., serotonin, BDNF, and tryptophan), contributing to lowered self-esteem, elevated feelings of isolation, and an increased risk of depression (29). Moreover, surveys of Chinese adults conducted during the pandemic indicate that individuals who participated in less PA were more susceptible to stress, whereas those who maintained home-based exercise routines reported benefits in coping with pandemic-related adversities (42, 43).

Collectively, exercise patterns from 2020 to 2022 underwent a notable decline and transformation, affecting cardiovascular health, immune function, obesity/metabolic issues, and psychological well-being. As societies adapt to a post-pandemic environment, further research and policy initiatives are needed to restore or maintain adequate activity levels, thereby mitigating long-term repercussions on physical and mental health.

## 5 Exercise patterns after the pandemic (2022–2025)

By 2023, most Asian countries lifted strict COVID-19 restrictions, ushering in a post-pandemic era characterized by a gradual return to normalcy. During this period, global PA levels partially rebounded. In Asia, regular PA among both younger and older populations not only recovered but also began to surpass pre-pandemic levels (25, 28), accompanied by lasting changes in individual exercise modalities.

### 5.1 Recovery of activity levels

With the reopening of gyms, parks, and sports facilities, many individuals have eagerly resumed their pre-pandemic exercise routines. Population surveillance data indicate that PA levels have rebounded from pandemic lows. For instance, a survey of older adults in Hong Kong compared activity levels between early 2022 (during a severe COVID-19 Pandemic) and late 2022 (after the Pandemic). The proportion of seniors classified as low-activity nearly halved from 45.9% to 28.6%, and their weekly exercise volume approximately doubled post-lockdown (44). Similar trends were observed in other demographics. In South Korea, national fitness data revealed that the prevalence of adequate PA, which remained stable pre-2020 but declined sharply during 2020-2021, began to normalize in 2022. Nevertheless, a complete return to pre-pandemic activity levels has yet to be achieved, as some individuals remain less active than they were before 2020 (28, 45). Public health experts emphasize the need for ongoing efforts to re-engage those who became inactive during the pandemic (46).

### 5.2 Enduring mode shifts and hybrid models

Although traditional exercise venues and group sports are experiencing a resurgence, the pandemic has left a lasting impact on exercise modalities. A hybrid fitness model has emerged, with many individuals incorporating home-based or digital workouts into their traditional gym sessions. Industry trend data reflect this shift: the popularity of home-based exercise surged during lockdowns, and although it has tapered as in-person options returned, it has not disappeared entirely. The American College of Sports Medicine's worldwide survey of fitness trends reported that “home exercise gyms” dropped from the second most popular trend in 2022 to the thirteenth in 2023, indicating a sustained interest in home fitness routines (47). Additionally, online fitness classes and coaching, which were relatively uncommon pre-COVID-19, have become regular components of many individuals' exercise regimens. Urban populations in Asia often blend physical gym attendance with online platforms, increasing the flexibility of both formats (32, 48).

### 5.3 Impacts on physical and psychological health

The resurgence of regular PA and the adoption of diverse exercise modalities in the post-pandemic period have yielded notable improvements in both physical and psychological health. The reinstatement of regular PA positively influences cardiovascular health. Increased engagement in both outdoor and indoor exercise has been associated with reduced blood pressure, improved lipid profiles, and enhanced overall cardiovascular function. This aligns with pre-pandemic evidence highlighting the cardioprotective effects of consistent PA (29, 34). Regular PA bolsters immune function, reduces susceptibility to infections, and aids recovery. Post-pandemic, the resumption of exercise routines has likely contributed to enhanced immune responses across populations, aligning with studies that emphasize the immune-boosting benefits of consistent PA (36–38). The return to regular exercise has played a crucial role in addressing pandemic-induced weight gain and metabolic disturbance. Increased PA levels have been linked to weight loss, improved insulin sensitivity, and better management of metabolic conditions, counteracting the adverse effects observed during periods of reduced activity (29, 34).

The revival of social and outdoor physical activities significantly alleviated depression and loneliness symptoms. Engaging in group exercises and spending time in natural environments are associated with enhanced mood and psychological resilience. Studies have shown that outdoor exercise not only prevents chronic diseases but also improves mental health by reducing anxiety and depression (29, 37, 39–41). Moreover, the availability of diverse exercise options, including indoor activities, has provided individuals with more opportunities to maintain regular PA, further supporting their mental health (42, 43).

In summary, the post-pandemic era has witnessed a resurgence in PA levels and the adoption of hybrid exercise models, leading to significant improvements in physical and psychological health outcomes.

## 6 Discussion

The findings indicate that prior to COVID-19, Asia was experiencing rising PA participation due to greater fitness awareness and better exercise infrastructure, although inactivity remained high among some groups (e.g., affluent youth). The pandemic abruptly reversed this trend, as lockdowns and gym closures caused a sharp drop in traditional exercise, shifting activity to home-based workouts and virtual classes. This change has contributed to adverse physical and mental health outcomes, mirroring global trends. However, the severity of restrictions and public responses varied widely across Asian countries, reflecting diverse policies and digital readiness. By 2023, many Asian populations saw a robust resurgence in activity, with evidence that PA levels exceeded pre-pandemic baselines. This contrasts with slower recoveries in certain Western countries, where activity levels remained below the 2019 baseline well into 2021 (49, 50).

Asia's relatively swift adaptation may be explained by the rapid adoption of hybrid fitness models (combining online and in-person exercise). In line with Self-Determination Theory, such formats fulfill autonomy (flexible scheduling), competence (progress tracking), and relatedness (virtual group classes), thereby sustaining participants' motivation to stay active (51, 52). Digital platforms also lower barriers (e.g., commute time and facility access), broadening exercise accessibility. These factors likely helped maintain exercise adherence despite the pandemic disruptions. Physiologically, moderate exercise is known to enhance immune function by increasing immunosurveillance through increased natural killer cells and anti-inflammatory cytokines, while prolonged sedentariness undermines immunity (38, 53). These benefits underscore the importance of restoring regular activities as societies recover.

Despite these positive trends, several limitations must be acknowledged. Asia's significant sociocultural and economic diversity means that PA patterns and pandemic-related impacts varied widely across different countries and subpopulations. Rural and underserved regions often face additional challenges, including limited access to digital infrastructure, healthcare resources, and organized exercise facilities, which may have further influenced PA engagement during and after the pandemic. Furthermore, most of the data synthesized in this review were drawn from secondary cross-sectional studies, limiting causal inference and generalizability. In particular, comprehensive peer-reviewed data specifically addressing rural populations remain scarce. Therefore, future longitudinal and region-specific studies are essential to better capture long-term behavioral changes and inform more targeted public health interventions across diverse Asian contexts.

## 7 Conclusion

**Figure 1:**
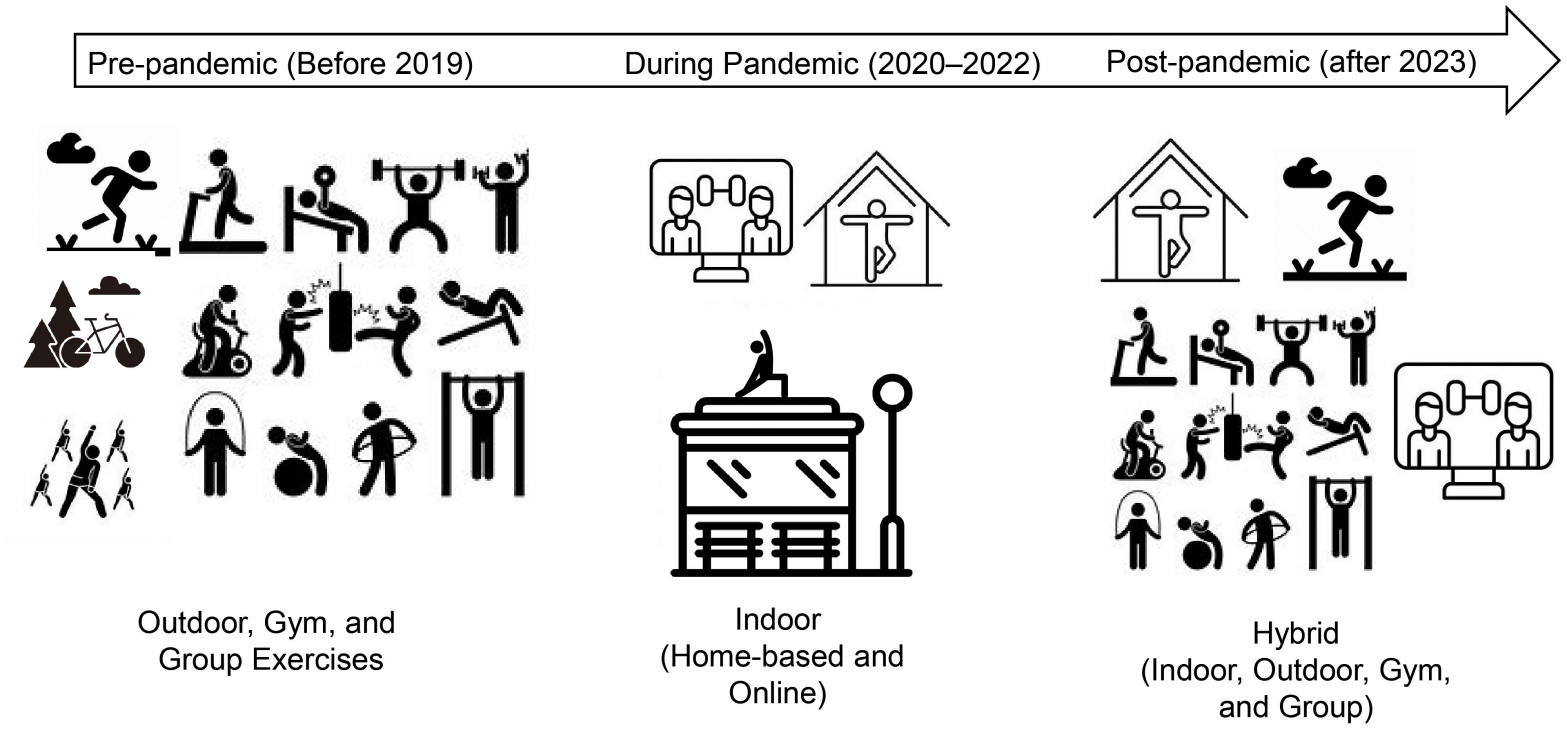
Evolution of physical activity patterns before, during, and after the COVID-19 pandemic. The figure illustrates the transition in predominant exercise modes across three distinct periods: pre-pandemic (before 2019), characterized by outdoor, gym-based, and group exercises; during pandemic (2020–2022), marked by a significant shift toward home-based indoor and online virtual exercise due to lockdown measures; and post-pandemic (after 2023), exhibiting a hybrid exercise pattern integrating indoor, outdoor, gym-based, group, and online activities.

The COVID-19 pandemic has underscored the critical role of PA in maintaining population health. While initial restrictions disrupted established exercise routines and shifted activity toward home-based and individualized formats, subsequent recovery demonstrated population resilience and adaptability through the widespread adoption of hybrid exercise models (Figure 1). These flexible, digitally enabled approaches offer diverse and accessible PA options, accommodating various preferences and lifestyles, particularly for individuals favoring home-centered routines.

Building on these adaptations, public health strategies should proactively integrate lessons learned from the pandemic into emergency preparedness and long-term health promotion. Policymakers and health organizations are encouraged to expand digital fitness offerings, particularly in rural and underserved regions where access remains limited, and to incorporate engagement features that promote sustainable adherence. Interventions should also be culturally tailored, leveraging Asia's strong tradition of group-based exercise formats to better engage youth, older adults, and community-based participants is vague in PA. Such targeted efforts will help strengthen community resilience and foster sustainable public health improvements in Asia's diverse populations.
